# Pre-exposure to Ambiguous Faces Modulates Top-Down Control of Attentional Orienting to Counterpredictive Gaze Cues

**DOI:** 10.3389/fpsyg.2020.02234

**Published:** 2020-09-09

**Authors:** Abdulaziz Abubshait, Ali Momen, Eva Wiese

**Affiliations:** ^1^ Robotics Domain, Italian Institute of Technology, Genoa, Italy; ^2^ Department of Psychology, George Mason University, Fairfax, VA, United States

**Keywords:** human-robot interaction, social-cognition, gaze-cueing, cognitive conflict, categorical boundary, mind perception

## Abstract

Understanding and reacting to others’ nonverbal social signals, such as changes in gaze direction (i.e., gaze cue), are essential for social interactions, as it is important for processes such as joint attention and mentalizing. Although attentional orienting in response to gaze cues has a strong reflexive component, accumulating evidence shows that it can be top-down controlled by context information regarding the signals’ social relevance. For example, when a gazer is believed to be an entity “with a mind” (i.e., mind perception), people exert more top-down control on attention orienting. Although increasing an agent’s physical human-likeness can enhance mind perception, it could have negative consequences on top-down control of social attention when a gazer’s physical appearance is categorically ambiguous (i.e., difficult to categorize as human or nonhuman), as resolving this ambiguity would require using cognitive resources that otherwise could be used to top-down control attention orienting. To examine this question, we used mouse-tracking to explore if categorically ambiguous agents are associated with increased processing costs (Experiment 1), whether categorically ambiguous stimuli negatively impact top-down control of social attention (Experiment 2), and if resolving the conflict related to the agent’s categorical ambiguity (using exposure) would restore top-down control to orient attention (Experiment 3). The findings suggest that categorically ambiguous stimuli are associated with cognitive conflict, which negatively impact the ability to exert top-down control on attentional orienting in a counterpredicitive gaze-cueing paradigm; this negative impact, however, is attenuated when being pre-exposed to the stimuli prior to the gaze-cueing task. Taken together, these findings suggest that manipulating physical human-likeness is a powerful way to affect mind perception in human-robot interaction (HRI) but has a diminishing returns effect on social attention when it is categorically ambiguous due to drainage of cognitive resources and impairment of top-down control.

## Introduction

In social interactions, we use information from social cues like gestures, facial expressions, and/or gaze direction to make inferences about what others think, feel, or intend (Adolphs, 1999; Emery, 2000; Gallagher and Frith, 2003). *Joint attention* or the ability to follow an interaction partner’s gaze in order to conjointly attend to an object of potential interest, is a fundamental social-cognitive mechanism that develops very early in life and is a precursor for higher-order social-cognitive processes, such as mentalizing or action understanding (for a review, see Frischen et al., 2007). In empirical research, joint attention can be investigated using the *gaze-cueing paradigm* (Friesen and Kingstone, 1998), where an abstract face stimulus is presented in the center of a screen that first looks straight at the participant and then changes its gaze direction to the left or right side of the screen (i.e., gaze cue), which is followed by a target that is presented either at the gazed-at location (i.e., valid trial) or opposite of the gazed-at location (i.e., invalid trial). This typically results in faster reaction times (RTs) to targets presented at valid than invalid locations (i.e., *gaze cueing effect*). Attentional orienting to gaze cues has traditionally been thought of as reflexive (i.e., a bottom-up process) as it is observable in infants as young as 3 months of age (Hood et al., 1998), is triggered by any kind of stimulus with eye-like configurations (Quadflieg et al., 2004), cannot be suppressed even when gaze direction is unlikely to predict the location of a target (i.e., *counterpredictive* cueing; Friesen et al., 2004; Vecera and Rizzo, 2006), and is not affected by a resource-demanding secondary task (Law et al., 2010). The few modulatory effects of gaze cueing that were originally reported were strongly dependent on participants’ age (i.e., stronger gaze cueing in children; Hori et al., 2005) and/or other individual traits (i.e., stronger gaze cueing in highly anxious individuals; Tipples, 2006; Fox et al., 2007).

More recently, however, studies have shown that attentional orienting to gaze cues *can* be top-down modulated when gaze cues are embedded in a richer context (the original experiments used abstract face stimuli; Friesen and Kingstone, 1998) that enhances the social relevance of the interaction for the observer (Tipples, 2006; Fox et al., 2007; Bonifacci et al., 2008; Graham et al., 2010; Kawai, 2011; Hungr and Hunt, 2012; Süßenbach and Schönbrodt, 2014; Wiese et al., 2014; Wykowska et al., 2014; Cazzato et al., 2015; Dalmaso et al., 2016, 2020; Abubshait and Wiese, 2017; Abubshait et al., 2020). Using such “social” versions of the original gaze-cueing paradigm, researchers were able to show that when social relevance is increased based on modulations of *similarity-to-self* (Hungr and Hunt, 2012; Porciello et al., 2014), *physical humanness* (Admoni et al., 2011; Martini et al., 2015), *facial expression* [Bonifacci et al., 2008; Graham et al., 2010; at long stimulus onset asynchrony (SOA) only], *social status* (Jones et al., 2010; Dalmaso et al., 2012, 2014, 2015; Ohlsen et al., 2013), *social group membership* (Dodd et al., 2011, 2016; Liuzza et al., 2011; Pavan et al., 2011; Ciardo et al., 2014; Cazzato et al., 2015; Dalmaso et al., 2015), and *familiarity* (Frischen and Tipper, 2006; Deaner et al., 2007) larger gaze cueing effects were observed (Wiese et al., 2013). Taken together, these findings suggest that engagement in joint attention may strongly depend on social context information, as well as a link between higher-level mechanisms of social cognition related to mentalizing, empathizing, or group membership and lower-level mechanisms of social cognition, such as joint attention (see Capozzi and Ristic, 2018 and Dalmaso et al., 2020, for comprehensive reviews on social factors that influence social attention).

With regard to human-robot interaction (HRI), potentially one of the most powerful contextual factors is the degree to which a robot is perceived to have a mind, with the ability to experience internal states like emotions and intentions and to execute goal-directed actions (i.e., *mind perception*; Gray et al., 2007). Seeing minds in others is not exclusive to humans, but “mind” can also be ascribed to agents that by definition do not have minds (e.g., robots) or whose mind status is ambiguous (e.g., animals; Gray et al., 2007). Mind perception is an automatic process that can be triggered implicitly when agents possess human-like *facial features* (Balas and Tonsager, 2014; Deska et al., 2016) or *behaviors* (Castelli et al., 2000). Decisions as to whether an agent “has a mind” are made within a few 100 ms (Wheatley et al., 2011; Looser et al., 2013), and just passively viewing stimuli that trigger mind perception is sufficient to activate social-cognitive brain networks (Wagner et al., 2011), even if their mind status is irrelevant to the task at hand (Wykowska et al., 2014; Caruana et al., 2015, 2017a). Mind status can also be explicitly ascribed to nonhuman agents when the presence of a human is needed in the current situation or when an entity has become so important to an individual that a “machine” status is no longer sufficient. For instance, agents of ambiguous physical human-likeness are more likely treated as a “human” when individuals are in an increased need of social contact due to chronic loneliness (Hackel et al., 2014) or when participants have to collaborate with them on a joint task (Hertz and Wiese, 2017). Likewise, soldiers who work with search-and-rescue robots on a regular basis are reported to be reluctant to agree to install updates on their robot “companions” because they fear this would change their “personality” (Singer et al., 2008; Carpenter et al., 2016).

Despite being an important question, studies investigating the effect of mind perception on social attention are surprisingly rare and have yielded mixed results depending on how mind perception was manipulated (Teufel et al., 2010; Wiese et al., 2012, 2014; Wykowska et al., 2014; Martini et al., 2015; Abubshait and Wiese, 2017). When mind perception was manipulated *via belief* (e.g., participants are instructed that changes in a robot’s gaze direction are pre-programmed vs. human-controlled), attentional orienting to gaze cues was enhanced when observed gaze behavior was believed to be caused by a human agent as opposed to a pre-programmed algorithm (Wiese et al., 2012; Wykowska et al., 2014; Caruana et al., 2015). Belief manipulations can also impact participants’ perceptions of the space around them (Müller et al., 2014; Fini et al., 2015), their performance in a joint Simon task (Müller et al., 2011), and their neural responses, as measured by activation in social regions of the brain (Kühn et al., 2014). A similar positive effect was found when mind perception was manipulated *via behavior* (e.g., predictive vs. random gaze cues), such that larger gaze cueing effects were observed when gaze cues predicted the target location with high likelihood as opposed to being non-predictive (80 vs. 50% predictive; Abubshait and Wiese, 2017). However, when mind perception was manipulated *via* physical *appearance* (e.g., gazers of varying degrees of human-likeness), results were more mixed: on the one hand, general differences in gaze cueing mechanisms were found between human and robot agents when using non-predictive cues (Admoni et al., 2011; Wiese et al., 2012), such that robots tended to induced smaller gaze cueing effects than humans when non-predictive gaze cues were used (i.e., 50% predictive of target location); this effect, however, was not further modulated by the robot’s physical human-likeness (Admoni et al., 2011; Martini et al., 2015; Abubshait and Wiese, 2017). On the other hand, a gazing stimulus that possesses very human-like but not perfectly human physical appearance (i.e., morphed images consisting of 70% of a human image and 30% of a robot image) disrupted top-down control of attentional orienting in counterpredictive gaze-cueing paradigms (i.e., targets appear with a higher chance at the uncued location), such that participants were less capable of shifting their attention away from the cued (i.e., not very likely target location) to the predicted (i.e., very likely target location) location when the gazer displayed ambiguous levels of human-likeness, as opposed to an unequivocally “human” or “robot” gazer (Martini et al., 2015).

The assumption that observing stimuli of ambiguous physical human-likeness negatively impacts resource-demanding cognitive processes, such as top-down control of attention, is in line with established biased-competition models of visual processing (Desimone and Duncan, 1995), showing that possible interpretations of ambiguous stimuli compete for representation in visual networks causing cognitive conflict and that cognitive resources are needed to direct selective attention to stimuli features that favor one explanation over the others (*via* inhibition of alternative category representations) to resolve the cognitive conflict (Meng and Tong, 2004; Sterzer et al., 2009; Ferrey et al., 2015). It is also in line with empirical examinations of the uncanny valley (UV; Mori, 1970) theory that links negative evaluations and long categorization times for ambiguously human-like face stimuli to categorical uncertainty (Cheetham et al., 2011; Hackel et al., 2014; Martini et al., 2016) and consumption of additional cognitive resources compared to categorically unambiguous stimuli (Wiese et al., 2019). Specifically, it was shown that when mind perception was manipulated *via* physical parameters, for instance, by morphing human images into robot images along a spectrum ranging from 0 to 100% of physical humanness, changes in mind ratings attributed to the resulting images show a categorical pattern, with significant changes in ratings at the human-nonhuman category boundary located at around 63% physical humanness, but only marginal changes in mind ratings for stimuli that unequivocally fall into either the “human” or “nonhuman” category (Cheetham et al., 2011, 2014; Hackel et al., 2014; Martini et al., 2016).

Follow-up studies showed that this qualitative change in mind ratings for stimuli located at the category boundary is associated with increased categorization times, indicating that being exposed to categorically ambiguous stimuli might be associated with increased cognitive processing costs compared to categorically unambiguous stimuli (Cheetham et al., 2011, 2014).

In support of this notion, a follow-up study used mouse tracking (Freeman and Ambady, 2010) to show that this increase in categorization time for stimuli of ambiguous human-likeness is associated with an increase in cognitive conflict, as indicated by larger mouse curvatures for stimuli of ambiguous human-likeness than unequivocally “human” or “robot” stimuli (Weis and Wiese, 2017; Wiese and Weis, 2020). Yet, another follow-up study showed that processing categorically ambiguous stimuli is also associated with an increase in cognitive costs and draining of cognitive resources over time even when the stimuli were irrelevant to the immediate task (Wiese et al., 2019). Specifically, the authors embedded face stimuli of differing levels of human-likeness (0% human, 30% human, 70% human, and 100% human) into a vigilance task, known to be sensitive to the drainage of cognitive resources (Parasuraman et al., 2009), and examined whether a categorically ambiguous stimulus of 70% physical humanness would be associated with a stronger decrease in performance over time (i.e., vigilance decrement) than a categorically unambiguous stimulus of 0, 30, or 100% physical humanness. In line with this assumption, the researchers showed that the 70% human stimuli caused a significantly larger decrement than the 0, 30, and 100% human stimuli, indicating that categorically ambiguous stimuli may drain more cognitive resources over time than categorically unambiguous stimuli, even when being irrelevant to the task (Wiese et al., 2019).

Interestingly, the negative effect on cognitive performance vanished for ambiguous stimuli when participants were perceptually pre-exposed to the stimuli before the task (i.e., both the ambiguous and unambiguous stimuli) by being asked to evaluate the stimuli regarding their capacity of having internal states (i.e., explicit mind perception; e.g., “*Can the stimulus feel pain*?”) or their perceptual features (i.e., implicit mind perception; e.g., “*Does the stimulus have the shape of an avocado*?”; Wiese et al., 2019). This suggests that cognitive conflict, when assessing the mind status of stimuli, is triggered by bottom-up mechanisms related to ambiguous perceptual stimulus features (Gao et al., 2010; Wheatley et al., 2011) and the automatic coactivation of competing categories (Ferrey et al., 2015), which can only be resolved by focusing selective attention on a subset of perceptual features that support one category over another – for instance, by pre-exposing participants to the stimuli and directing attention to their perceptual features. Regarding attentional orienting to gaze signals, this means that manipulating the degree to which a gazer is perceived to “have a mind” *via* physical features can have negative consequences on the effectiveness of a gaze cue when the gazer is of ambiguous physical human-likeness, which could drain cognitive resources and negatively impact top-down control of spatial attention.

### Aim of Study

The goal of the current study is to investigate whether a perceptually ambiguous agent induces cognitive conflict due to an increased difficulty in categorizing a face stimulus as “human” or “nonhuman” (Experiment 1), whether the categorically ambiguous face can potentially interfere with top-down control of attentional orienting to gaze cues due to cognitive conflict (Experiment 2), and whether resolving perceptual ambiguity *via* pre-exposing participants to the stimuli prior to the gaze-cueing task would restore top-down control abilities (Experiment 3). To investigate these questions, we created stimuli of varying degrees of physical humanness – ranging from 0 to 100% human image contained in the morphed image in steps of 20% – and embedded them into a mouse-tracking task (Experiment 1) and a gaze-cueing task (Experiments 2 and 3). The mouse-tracking task is a force-choice categorization task that is designed to investigate the coactivation of two competing categories with larger overlap in coactivation correlating with larger cognitive conflict, as measured by mouse-movement curvatures (Freeman and Ambady, 2010). In the gaze-cueing task, we used a counterpredictive paradigm where participants responded to targets that appeared more often at the uncued location (80% of trials) than the cued location (20% of trials), to disentangle bottom-up from top-down mechanisms (Friesen et al., 2004). In order to optimize task performance, participants have to suppress bottom-up attentional orienting to the cued (but unlikely) target location and, instead, shift their attentional focus to the uncued (but likely) target location *via* top-down control. If attentional orienting to gaze cues follows a bottom-up pattern, reaction times will be shorter for valid than invalid trials (i.e., a standard gaze cueing effect: invalid minus valid trials reaction time difference is *positive*); if attentional orienting follows a top-down pattern, reaction times should be shorter for invalid than valid trials (i.e., a reversed gaze cueing effect: invalid minus valid trials reaction time difference is *negative*). Thus, in counterpredictive gaze-cueing paradigms, the difference in reaction times between invalid and valid trials can be used as a measure for the extent to which gaze cueing is top-down controlled: the more positive (negative) the difference in reaction times, the more pronounced the bottom-up (top-down) component is.

If mind perception caused cognitive conflict for stimuli located at the category boundary between “human” and “nonhuman” (located at around 60–70% physical humanness, as indicated by previous work; Cheetham et al., 2011, 2014; Wiese et al., 2019; Wiese and Weis, 2020), we would expect that the 60% human morph^1^ would induce the most cognitive conflict that is due to categorization compared to faces that are easily distinguished as “human” or “nonhuman” (Experiment 1). This cognitive conflict should also significantly disrupt top-down control of attentional orienting for the morphed face that showed the most cognitive conflict (Experiment 2). Furthermore, top-down control should be restored when participants are pre-exposed to the stimuli’s perceptual features prior to the gaze-cueing task (Experiment 3).

## Experiment 1

The aim of Experiment 1 was to use mouse tracking to measure if perceptually ambiguous faces caused cognitive conflict that is due to categorizing the faces as “human” or “nonhuman” *via* measures of mouse curvatures and to identify which of the faces was closest to the category boundary. If, indeed, categorically ambiguous faces induced conflict due to categorization, we would expect that mouse curvatures should be largest for the 60% morphed human face as previous literature suggests that the category boundary exists around the 60% physical humanness level (Cheetham et al., 2011, 2014; Hackel et al., 2014; Martini et al., 2016).

### Materials and Methods

#### Participants

Thirty-eight participants were recruited from the George Mason University undergraduate pool (25 females, *M* age = 20.68, *SD* = 4.07, range = 18–35). Students were given course credit for completion of the study. All participants reported normal or corrected-to-normal vision and provided written consent prior to participating. All research procedures were approved by George Mason University’s Internal Review Board. All data and analysis scripts can be found on https://osf.io/73pr6/.

#### Stimuli

**Figure 1:**
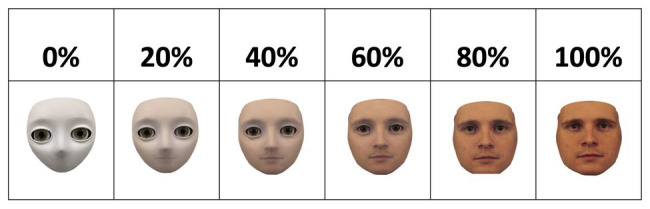
Spectrum of physical humanness ranging from 0 (left) to 100% (right) of physical humanness. Morphed face stimuli were created by morphing the face image of a humanoid robot into the image of a male human face from the Karolinska Directed Emotional Faces (KDEF) database (Lundqvist et al., 1998). The morphed images increase in physical humanness from the left side of the spectrum (i.e., robot) to the other (i.e., human) in increments of 20%.

The face stimuli were created using FantaMorph, a software that allows two images to be morphed into one another incrementally, resulting in a spectrum ranging from 0% of image A (i.e., 100% of image B) to 100% of image A (i.e., 0% of image B). On the “nonhuman” end of the spectrum, the S2 humanoid robot head developed by Meka Robotics was used. On the “human” end of the spectrum, a male face stimulus from the Karolinska Directed Emotional Faces (KDEF) database was used (Lundqvist et al., 1998). The spectrum comprised of six morph levels, resulting in stimuli of 0, 20, 40, 60, 80, and 100% physical human-likeness; see Figure 1.

#### Task and Procedure

**Figure 2:**
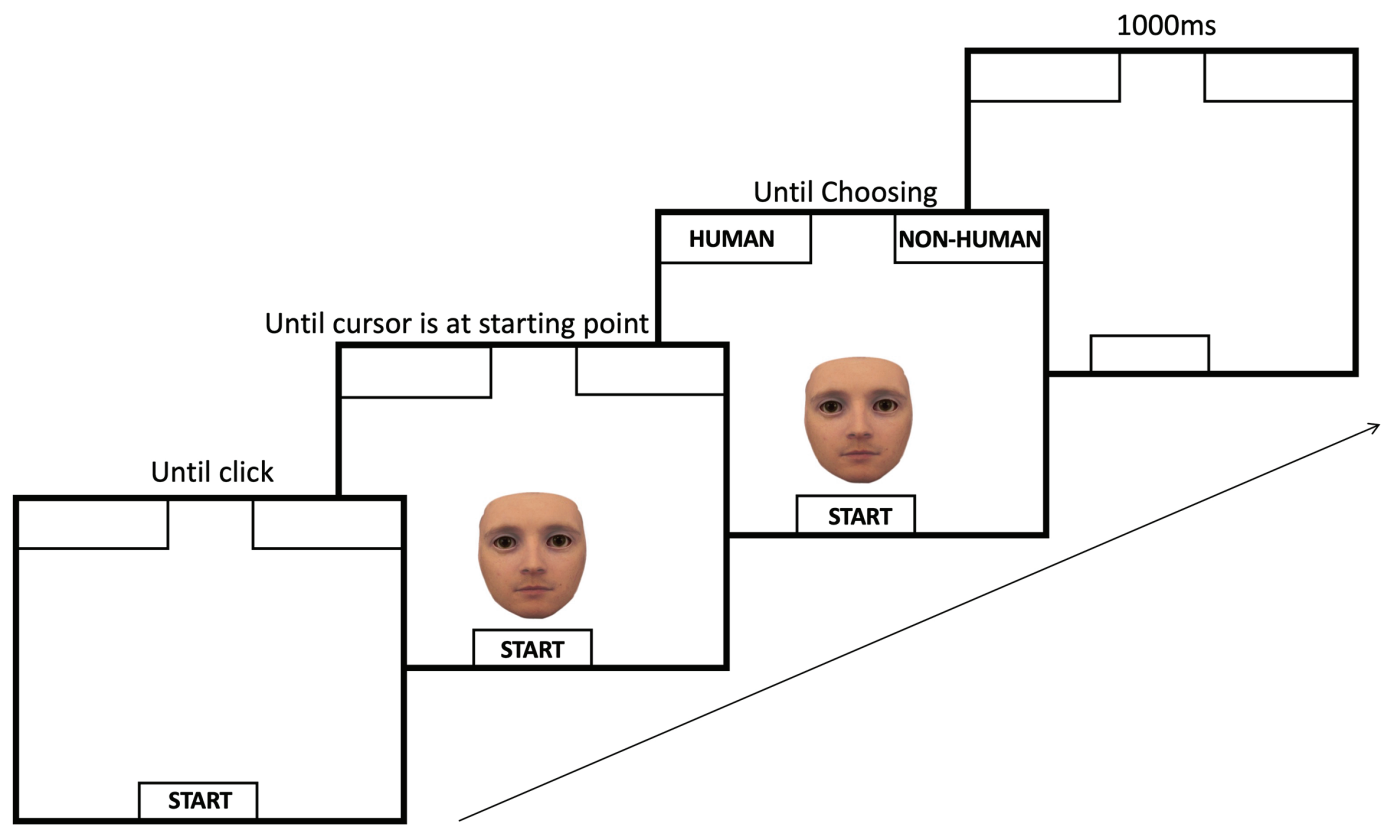
Sequence of events during a trial of mouse tracking. On each given trial, participants moved their mouse cursor to the start position at the bottom of the screen and clicked the start button. After the click, a face would be presented centrally at the bottom of the screen, right above the start button. Immediately after the face is presented, the category labels appeared. Next, the participant moved the mouse to pick one of the categories. The inter-trial-interval (i.e., ITI) was set to 1,000 ms.

Stimuli were presented (one at a time) at the bottom center of the screen and asked participants to categorize them as either “human” or “nonhuman” by moving their mouse cursor to the respective labels presented in the top left or top right corner of the screen as soon as the image appeared on the screen. The location of the labels was counterbalanced across participants. At the beginning of each trial, participants were asked to move the mouse cursor to a designated starting position, which was located centrally at the bottom of the computer screen, and to click the left mouse button to initiate the trial. Immediately afterward, one of the morphed stimuli was presented centrally at the bottom of the screen, and participants had to move the mouse cursor from the starting position to one of the two category labels located in the top two corners of the screen and click the label. For each morphed image, mouse cursor movement trajectories were measured. After each trial, participants were presented with a blank black screen for an inter-trial interval (ITI) of 1,000 ms to signify the end of the trial; see Figure 2.

Participants were instructed to complete the task as quickly as possible to maximize the likelihood of the mouse moving from the starting position and to limit the number of trials where participants would keep the mouse stationary and only move it once they have categorized the face. We did not want participants to notice that the faces formed a spectrum that progressed systematically in degree of human-likeness to account for any bias that could correspond to the pattern. Therefore, faces were presented to participants in a randomized fashion. Additionally, to further conceal the pattern among the faces, eight decoy human-robot spectrums (that were created with the same procedures) were included among the stimuli. Each experimental session started with a practice block in which participants completed three trials with three different morphed agents that were not included in the main task. After completing the practice block, participants moved to the experimental condition in which they saw all 54 faces in a randomized fashion. Each face was presented once for a total of 54 trials. The study took approximately 20 min to complete.

#### Analysis

**Figure 3:**
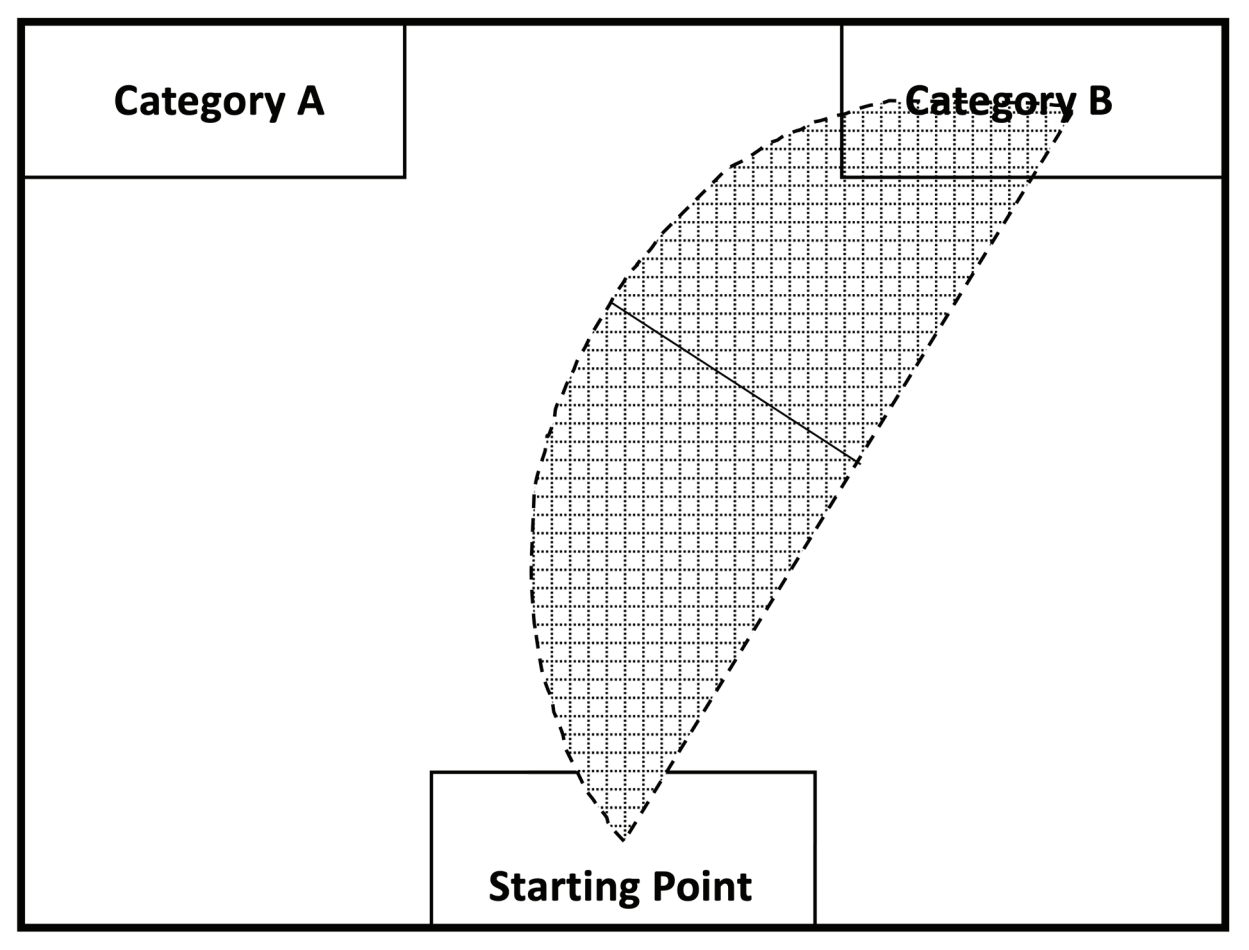
Mouse tracking recording and analysis (adapted from Freeman and Ambady, 2010). The shaded region visualizes how mouse curvature is used to calculate the area under the curve (AUC): the curved line represents the participant’s actual mouse trajectory while picking a category; the straight line represents the theoretical mouse trajectory if no cognitive conflict between the indicated categories occurs for a given stimulus. A comparison is, then, drawn between the maximal deviation of the actual mouse movement and the theoretical straight line to calculate the AUC (i.e., solid black line).

Data were analyzed using R (version 3.6.1). The mouse-tracking software developed by Freeman and Ambady (2010) was used to collect and process mouse tracking data. The software allows researchers to record time-standardized trajectories of the mouse’s movements for a given trial. This allows users to compute the area under the curve (AUC), which is the geometric area of the mouse trajectory from the mouse’s starting point to the end point compared to a straight line trajectory from those points (Freeman and Ambady, 2010). When participants are conflicted between two choices regarding a stimulus, an overlap in activation between the two categories would cause participants to make a choice in a geometrically wide mouse movement, which would result in a large AUC; a stimulus that does not coactivate two categories should induce less conflict and result in a geometrically narrow movement and a small AUC. The general idea underlying mouse tracking is depicted in Figure 3. None of the mouse-tracking trials deviated more than 3 *SD* from the participant’s mean and were all kept in the analysis.

**Figure 4:**
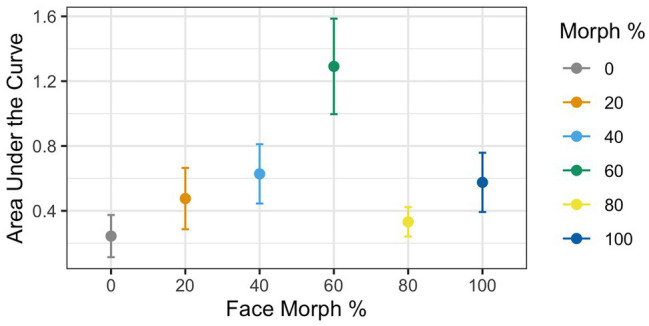
AUC during mouse tracking as a function of physical human-likeness. The 60% human morph is associated with a significantly stronger cognitive conflict than all the other morphs combined.

To analyze the mouse tracking data, a univariate ANOVA with AUC as a dependent variable and *Agent Type* as a within-participants factor (0% human vs. 20% human vs. 40% human vs. 60% human vs. 80% human vs. 100% human) was conducted. Follow-up *t*-tests were corrected using the false discovery rate (FDR) procedure.

### Results

Greenhouse–Geisser corrections were done (*ε* = 0.65) due to violation of the sphericity assumption according to the Mauchly test [*χ* ^2^(6) = 0.22, *p* < 0.001]. Results revealed a significant main effect of *Agent Type* [*F*(1,37) = 3.83, *p* = 0.009, *η_G_* ^2^ = 0.08], with mouse curvatures varying as a function of physical human-likeness. To examine whether mouse curvatures were more pronounced for the 60% morph than the other stimuli, we used contrast coding comparing the average mouse curvatures for the 60% morph to the grand mean of all other face stimuli. This analysis revealed a significant difference between the 60% face and the grand mean of all the other morph stimuli [*t*(37) = 4.03, *p* < 0.001, *d* = 0.57], such that the 60% morph had a significantly higher AUC compared to the average AUC of all the other faces (AUC: *M*_60% face_ = 1.29 vs. *M*_Grand_ = 0.45; see Figure 4). This suggests that the 60% morph was perceived as categorically more ambiguous and that it potentially triggered larger cognitive conflict compared to the other face stimuli.

### Discussion

Experiment 1 aimed to use an established technique for measuring cognitive conflict processing, namely mouse tracking, to examine if categorically ambiguous faces induced cognitive conflict that is due to categorizing them as a “human” or “nonhuman” and if the faces that have been previously shown to be close to the category boundary (i.e., 60% humanness) induced the most cognitive conflict. As such, we expected to find that the 60% human face would exert the most cognitive conflict. Results of Experiment 1 showed that, indeed, the level of morphing had an overall effect on cognitive conflict and that the supposedly categorically ambiguous 60% morph induced significantly more cognitive conflict than all of other morphed images together (as measured in AUC) when subjects were categorizing the faces as a human or nonhuman.

## Experiment 2

Experiment 2 aimed at examining whether the category boundary face (i.e., the face that exerted the most cognitive conflict in Experiment 1) has the ability to disrupt top-down modulation of attention orienting compared to faces that are more easily distinguishable as either a human or a nonhuman. If perceptual ambiguity drained cognitive resources, less cognitive resources would remain for an observer to exert top-down modulation of attentional orienting (i.e., attending to the predicted location as opposed to the cued location). As such, we would expect to find significant differences in gaze cueing for categorically ambiguous than non-ambiguous faces. Specifically, we expect the categorically ambiguous face (i.e., the 60% human face) to elicit stronger reflexive orienting of attentional resources (i.e., standard gaze cueing effect with shorter reaction times on valid trials) than categorically unambiguous faces as cognitive resources that would facilitate top-down modulation should be more depleted for categorically ambiguous face stimuli compared to unambiguous faces.

### Materials and Methods

#### Participants

Thirty-seven undergraduate students from George Mason University were recruited to participate in the study (20 females, *M* age = 20.3, *SD* = 3.33, range = 18–37). All participants reported normal or corrected-to-normal vision and provided written consent as required and approved by the George Mason University Office Internal Review Board. Data from Experiment 2 were previously published (Martini et al., 2015) but was retreated, reanalyzed, and re-discussed from its original framework.

#### Stimuli

The stimuli used for Experiment 2 were identical to Experiment 1 with the addition of averted gaze stimuli. To create the averted gaze stimuli, we used Photoshop to shift irises and pupils of the original human and robot images by 0.4° from direct gaze. The target stimuli were black capital letters (F or T) presented at 0.5° high and 0.9° wide. Targets were presented on the same horizontal axis as the eyes of the respective stimulus and were located 14.7° to either the left or right from the center of the screen. All stimuli were 7.8° wide and 8.6° high and were depicted on a white background.

#### Task and Procedure

At the beginning of each session, participants were seated at a distance of 70 cm away from the computer monitor and were provided instructions to the gaze-cueing paradigm by a researcher. The instructions required them to fixate their gaze on a fixation cross in the middle of the screen and to respond to the identity of a target probe (“F” or “T”) as quickly and accurately as possible (i.e., discrimination task). Half of the participants were required to press the “K” key for the target letter “T” and the “D” key for the target letter “F”; key assignments for the targets were reversed for the other half of the participants.

Afterward, participants provided written consent and completed a practice block with 20 trials. The practice block included a mechanistic-looking robot as a gazer to insure that participants were not pre-exposed to the morphed stimuli prior to the experimental block. After the practice block, participants completed six experimental blocks – one block for each of the six morphed stimuli. Experimental blocks included 60 trials of counterpredictive cueing, in which the target appeared in the uncued location 80% of the time (i.e., 20% were valid and 80% were invalid trials). The order of the experimental blocks was randomized across participants to account for potential sequence effects associated with seeing the different morphed faces at different time points throughout the experiment. At the beginning of each experimental block, participants were presented with the stimulus and were asked to rate its mind status (i.e., *Do you think this agent has a mind?*) on an eight-point Likert scale ranging from 1 (“definitely not”) to 8 (“definitely yes”). Afterward, participants completed 60 trials of gaze cueing for this particular stimulus and then moved on to the next stimulus. This procedure was repeated until all blocks were completed.

**Figure 5:**
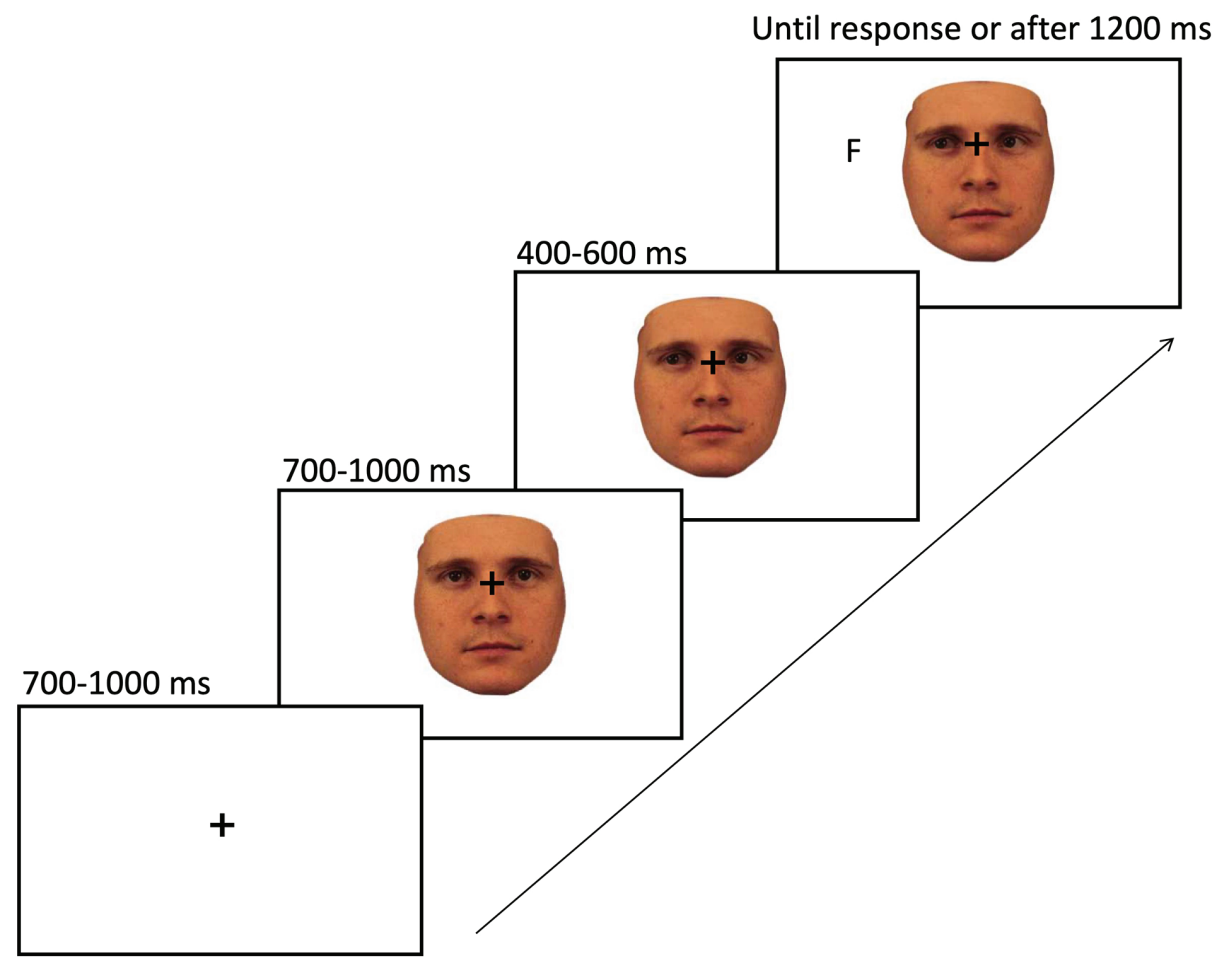
Sequence of events on a given trial of gaze cueing. The paradigm started with the presentation of a fixation cross in the center of the screen for 700–1,000 ms, followed by a face stimulus looking straight (here the 100% human face). About 700–1,000 ms later, the face would change its gaze direction to either the left or right side of the screen. After a stimulus onset asynchrony (SOA) of 400–600 ms, a target (F or T) would appear in either the same (i.e., valid trial) or opposite direction of the cue (i.e., invalid trial). The target letter remained on the screen until a response was given or a time out was reached, whichever came first.

The sequence of events on a given trial of gaze cueing is shown in Figure 5. At the beginning of each trial, a fixation cross was presented in the center of the screen and after a jittered interval of 700–1,000 ms, and one of the stimuli appeared in the center of the screen with the fixation cross still being visible. About 700–1,000 ms later, the face shifted its gaze direction to the right or left side of the screen, which constituted the gaze cue. After a SOA of 400–600 ms, the target letter (i.e., T or F) would appear on the screen, either in the cued location (i.e., valid trial; 20% of all trials) or in the opposite direction of the gaze cue (i.e., invalid trial; 80% of all trials). The target stayed on the screen until the participant responded by key press (“K” or “D” key) or a timeout criterion was reached after 1,200 ms, whichever came first. The time between the target appearing on the screen and the participant pressing the assigned key was recorded as reaction time for the analysis. The interval between trials (i.e., ITI) was set to 680 ms.

#### Analysis

For subjective mind ratings, a six-level univariate within-subject ANOVA with *Agent Type* as a factor (i.e., 0% human vs. 20% human vs. 40% human vs. 60% human vs. 80% human vs. 100% human) was used to examine differences in ratings between the faces. Two *post hoc t*-tests were used to test if people subjectively perceived the 60% human face differently than the 80% human face and the 60% human face differently than the 40% human face.

To analyze the gaze cueing data, average RTs were computed for each valid and invalid trial per face, per participant. Only correct trials were used to compute RT averages on the single participant level. A 2 × 6 within-participant ANOVA with *Validity* (i.e., valid vs. invalid) and *Agent Type* (i.e., 0% human, 20% human, 40% human, 60% human, 80% human, and 100% human) as factors was used to examine the effect of physical human-likeness on attentional orienting to gaze cues. All *post hoc t*-tests were corrected using the FDR procedure.

### Results

**Figure 6:**
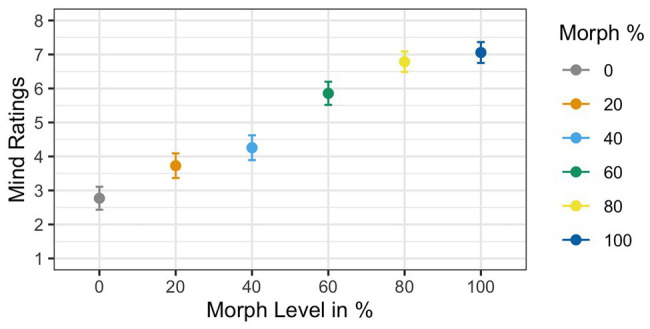
Mean mind ratings as a function of face morph. A main effect of agent type was found. Additionally, *post hoc t*-tests illustrated that the 60% human face was rated significantly different from both the 40% human and the 60% human faces.

Two participants were removed from the analysis for having accuracy rates below 85%, resulting in a sample size of 35 participants. Results of the ANOVA analyzing subjective mind ratings violated Mauchly’s test for sphericity [*χ* ^2^(6) = 0.21, *p* < 0.001]. Greenhouse–Geisser corrections were used to account for the assumption violation (GG *ε* = 0.65). Corrected estimates revealed a significant effect of *Agent Type* [*F*(1,34) = 53.11, *p* < 0.001, *η_G_* ^2^ = 0.39]. The main effect of mind ratings showed that mind ratings increased as faces increased in physical humanness; see Figure 6. *Post hoc t*-tests revealed that the 60% human face was significantly different from both the 40% human face [*t*(34) = 4.72, *p* < 0.001, *d* = 0.76] and the 80% human face [*t*(34) = −2.73, *p* < 0.01, *d* = −0.48].

**Figure 7:**
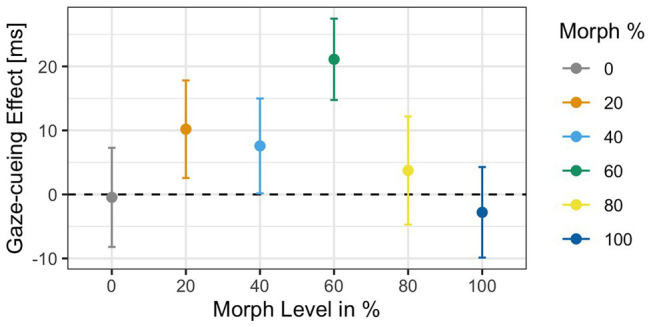
Reaction times as a function of physical human-likeness. Gaze cueing effects, which are calculated as the average reaction time differences between invalid and valid trials, are positive (indicative of reflexive attentional orienting to the cued location) for any of the “morphed” images but the “unmorphed” original 100% human and 100% robot faces, with the 60% morph showing the strongest “reflexive” cueing pattern. Together with the results of Experiment 1, these data indicate that there may be a direct link between the amount of cognitive conflict a face stimulus causes (and the amount of cognitive resources it drains) and the degree to which top-down control of attentional orienting to gaze cues is reduced.

Analysis of the RTs to targets in the gaze-cueing task showed that participants performed at a high level of accuracy as only 5% of the trials were rejected due to incorrect responses (*M* accuracy = 95%, *SD* = 0.03). Results of the 2 × 6 ANOVA revealed no significant main effect of *Agent Type* [*F*(1,34) = 0.32, *p* = 0.9, *η_G_* ^2^ = 0.002] but a significant main effect of *Validity* [*F*(1,34) = 5.21, *p* = 0.02, *η_G_* ^2^ = 0.001], with shorter reaction times on valid (*M* = 503 ms, *SD* = 91.1) than invalid trials (*M* = 510 ms, *SD* = 84.9), which indicated that attentional orienting followed a reflexive pattern on average; the *Agent Type* × *Validity* interaction [*F*(1,34) = 1.3, *p* = 0.26, *η_G_* ^2^ = 0.002] was not significant. The gaze cueing effects as a function of physical human-likeness are shown in Figure 7.

Since we had the directed hypothesis that the categorically most ambiguous stimulus (i.e., 60% morph) would diminish top-down control more strongly than categorically less ambiguous stimuli, we first compared RTs on valid vs. invalid trials for each level of physical human-likeness separately and then compared the average gaze cueing effect in response to the categorically ambiguous 60% morph to those of the categorically unambiguous morphs (all other morphs). Paired *t*-tests for each morph level separately showed that the RT-difference between valid and invalid trials was only significant for the 60% human morph [*t*(34) = −2.82, *p* = 0.03, *d* = −0.22], with significantly shorter RTs on valid than invalid trials (*M*_valid_ = 491 ms vs. *M*_invalid_ = 512 ms), which indicated that attentional orienting to a categorically ambiguous gazer followed a reflexive pattern (i.e., standard gaze cueing effect); whereas, the other faces did not significantly induce a reflexive pattern (i.e., RT-difference between valid and invalid trials was not significant): 0% human [*t*(34) = 0.06, *p* = 0.95, *d* < 0.01; *M*_valid_ = 509 ms vs. *M*_invalid_ = 508 ms], 20% human [*t*(34) = −1.36, *p* = 0.51, *d* = −0.12; *M*_valid_ = 505 ms vs. *M*_invalid_ = 516 ms], 40% human [*t*(34) = −1.01, *p* = 0.62, *d* = −0.07; *M*_valid_ = 500 ms vs. *M*_invalid_ = 508 ms], 80% human [*t*(34) = −0.5, *p* = 0.84, *d* = −0.03; *M*_valid_ = 509 ms vs. *M*_invalid_ = 513 ms], and the 100% human [*t*(34) = 0.37, *p* = 0.84, *d* = 0.03; *M*_valid_ = 506 ms vs. *M*_invalid_ = 503 ms]. Furthermore, contrast coding showed that the gaze cueing effect for the 60% human morph differed significantly from the gaze cueing effects for all other faces combined [*t*(34) = 2.11, *p* = 0.03, *d* = 0.42], with larger standard/reflexive gaze cueing effects for the 60% morph than all other morphs combined [gaze-cueing effect (GCE): *M*_60% face_ = 21.1 ms vs. *M*_Grand_ = 3.65 ms].

#### Exploratory Analysis

Although the results indicated that the 60% human morph induced a stronger cognitive conflict (Experiment 1), as well as stronger reflexive attentional orienting than any of the other morphed images (Experiment 2), the current analyses do not directly link cognitive conflict processing as measured *via* mouse tracking to the amount of reflexive attentional orienting to gaze cues. Since two experiments contained independent samples, a traditional correlation analysis linking gaze cueing to mouse curvatures is not possible. To address this issue, we calculated average gaze cueing effects (i.e., RTs on invalid trials − RTs on valid trials) and average AUC values for each morphed face stimulus across participants and calculated the correlations at the morph level. In doing so, the sample of interest is at the morph level (i.e., each morphed face now has one gaze-cueing effect and one AUC value) and not the participant level, which allows us to directly compare the two samples. The Spearman correlation analysis showed a significant correlation of 0.81 between AUC and GCE [*r*(4) = 0.81, *p* = 0.04], indicating that cognitive conflict measures, such as mouse tracking, are related to top-down control of social attention.

While the correlation was positive and significant, a traditional parametric correlation would not yield stable estimates if the sample size is not large enough. In other words, with a sample size of 6, parametric analyses do not yield reliable estimates due to violations of assumptions regarding normality. To alleviate this issue, we adopted a Bayesian approach as estimate interpretation is viable with a small sample when using Bayesian methods (e.g., McNeish, 2016). A Bayesian analysis would allow us to determine the strength of the evidence in support of our hypothesis that stronger cognitive conflict processing for a given stimulus would lead to stronger bottom-up processing of social attention (i.e., positive gaze cueing effects), as opposed to parametric analyses which determine, in a binary fashion, if we can or cannot reject the null hypothesis (i.e., does a relationship between cognitive conflict and gaze cueing effects exist?). If attentional orienting to gaze cues was, indeed, impacted by the amount of cognitive conflict associated with categorically ambiguous face stimuli, we would expect to find a positive relationship between gaze cueing effects and AUC (i.e., the higher the cognitive conflict triggered by a face stimulus, the higher the AUC, the more reflexive attentional orienting should be, which would be reflected in shorter RTs on valid than invalid trials).

**Figure 8:**
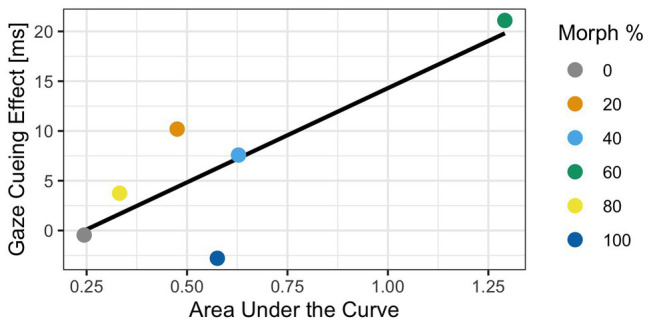
Results of the Bayesian analysis exploring the relationship between gaze-cueing effect (GCE) and AUC. The graph illustrates the positive relationship between GCE in Experiment 2 and AUC in Experiment 1 for each of the morphed stimuli. Specifically, as the AUC becomes larger, the GCE becomes larger too, indicating that the amount of cognitive conflict a face induces is correlated to the “reflexiveness” of gaze cueing. The blue line illustrates the line of best fit.

Before estimating the Bayesian correlation, we need to use a sampling procedure that allows us to draw estimates and their probabilities from a distribution. We used 5,000 iterations of the Markov chain Monte Carlo (MCMC) sampling procedure to determine the estimate of the correlation. Additionally, we used a uniform prior distribution to draw our estimates from. This allows us to test the strength of the relationship between gaze cueing effects and AUC. Results of the Bayesian correlation showed that a positive relationship between GCE and AUC [*r* = 0.68, 90% CI (0.13, 0.99)] with a 93% probability that the true correlation estimate is greater than zero and positive, which indicated that the relationship is likely. This suggests that there is enough evidence to confidently conclude a positive relationship between GCE and AUC; see Figure 8.

### Discussion

After identifying which face exerted the most cognitive conflict in Experiment 1, Experiment 2 aimed to examine whether the same categorically ambiguous face (i.e., 60% human face) would negatively impact top-down control of attentional orienting to gaze signals compared to unambiguous stimuli due to drainage of cognitive resources. Using a counterpredictive gaze-cueing paradigm allowed us to determine whether attentional orienting was volitionally shifted to the uncued but likely target location (i.e., faster reaction times on invalid vs. valid trials) or whether it was reflexively shifted to the cued but unlikely target location (i.e., faster reaction times on valid vs. invalid trials). We expected to find that the 60% human face would be subjectively different than the faces that are adjacent to it (i.e., the 40% human face and the 80% human face) and if it would be associated with stronger reflexive processing as shown by faster reaction times to the cued but unlikely direction for the category boundary face compared to the non-category boundary faces.

The results of the subjective ratings show that, as expected, the 60% human face was categorically different than the 40% human face (i.e., nonhuman) and the 80% human face, indicating that participants subjectively perceived the ambiguous face differently than the other faces. Additionally, the results show that variation of gaze cueing effects (i.e., RTs on invalid trials − RTs on valid trials) as a function of physical human-likeness follows an inverted u-shaped pattern, with negative or reversed gaze cueing effects for the “unmorphed” images, namely the 100% robot and the 100% human face. All “morphed” images (20–80% human morph) showed positive or standard gaze cueing effects to some extent, indicating that they reflexively oriented attention to some degree. Inline with our main hypothesis, the categorically ambiguous 60% human morph induced gaze cueing effects that are significantly more positive/reflexive than the other, categorically unambiguous, morphs combined. These results show that although the 60% morph (i.e., the categorically most ambiguous stimulus) impacted top-down control most negatively, it is possible that the other morphed stimuli created a certain level of categorical ambiguity that affected top-down control mechanisms to varying degrees, as determined by the positive relationship between gaze cueing effects and cognitive conflict that is due to categorization.

## Experiment 3

Experiments 1 and 2 illustrated that categorically ambiguous faces can induce cognitive conflict that may be due to increased cognitive costs invested in resolving perceptual conflict between two competing categories (“human” vs. “nonhuman”), and this influences observers’ ability to top-down control attentional orienting. However, based on their design, the experiments conducted so far are only able to show a correlational relationship between cognitive conflict associated with categorical ambiguity and a reduction in top-down control of attentional orienting. In order to show that cognitive conflict is *causal* to a modulation of top-down control, the amount of cognitive conflict a stimulus induces would have to be manipulated experimentally. To address this, we experimentally reduced the extent to which categorically ambiguous faces induced cognitive conflict by pre-exposing participants to the faces prior to the gaze-cueing task in Experiment 3. We, then, compared the size of the gaze cueing effects that they trigger from Experiment 3 to gaze cueing effects measured in Experiment 2. The assumption that pre-exposure would alleviate categorical ambiguity was based on previous reports that conflict processing on one trial leads to transient upregulation of selective attention on the next trial, which biases perception toward one particular interpretation and reduces cognitive conflict even without explicit instructions to pay attention to category-defining features (*conflict adaptation*; e.g., Botvinick et al., 2004; Egner and Hirsch, 2005). The effectiveness of perceptual pre-exposure to reduce categorical ambiguity and the associated drainage of cognitive resources has also been validated empirically by Wiese et al. (2019). Exposing participants to the stimuli during a perceptual task before the gaze-cueing task should speed up conflict adaptation by (a) explicitly paying attention to the perceptual features of the stimuli and (b) resolving the conflict without being forced to concurrently perform another task that requires resources. If pre-exposure resolved categorical ambiguity with the 60% morph, competing activation of the human and nonhuman categories should be reduced and free up resources for top-down control. Additionally, we were interested to see how the category boundary would be affected when people are pre-exposed to the faces.

### Materials and Methods

#### Participants

Thirty-four undergraduate students from George Mason University (24 females, *M* age = 22.12, *SD* = 5.10, range = 18–37) were recruited for the study. All participants reported normal or corrected-to-normal vision and provided written consent prior to participating. All research procedures were approved by George Mason University’s Internal Review Board.

#### Stimuli, Task, and Procedure

**Figure 9:**
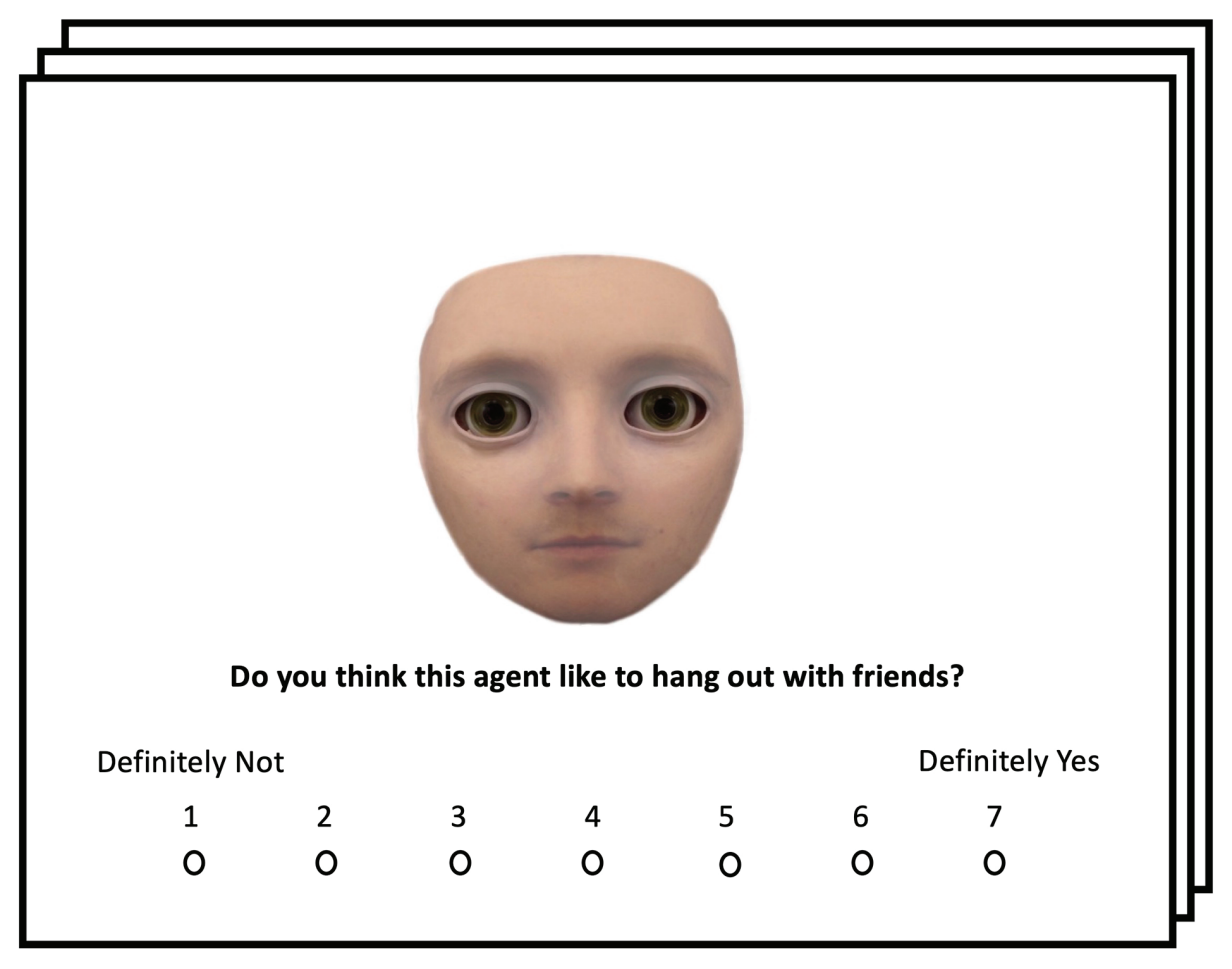
Example of a pre-exposure trial. Face stimuli were presented in random order and participants were instructed to rate the presented face regarding different mental capacities related to mind perception; see Wiese et al. (2019) for more details regarding the pre-exposure manipulation.

Stimuli used in Experiment 3 were identical to Experiments 1 and 2. The task and procedure were the same as Experiment 2 with one exception: before completing six blocks of gaze cueing with the six different face stimuli, participants completed a pre-exposure task during which they were asked to rate each face stimulus on an eight-point Likert scale (1 being “Definitely Not” and 8 being “Definitely Yes”) regarding five questions related to mind perception: “Please rate how much this face has a mind?,” “Please rate how much this face looks alive?,” “Do you think this agent would feel pain if it tripped and fell on hard ground?,” “Do you think this agent like to hang out with friends?,” and “Do you think this agent is an interesting conversationalist?” (Martini et al., 2016). These questions were chosen since they have been shown to significantly reduce cognitive conflict for categorically ambiguous face stimuli during pre-exposure (Wiese et al., 2019). All faces and questions were presented in a randomized fashion during pre-exposure. After having rated all face stimuli, participants completed an identical gaze-cueing paradigm to Experiment 1. An example trial from the pre-exposure is shown in Figure 9.

#### Analysis

Analyses in Experiment 3 were identical to Experiment 2, with one exception: we conducted an additional 2 × 2 mixed ANOVA for only the 60% human morph between participants in Experiment 2 and participants in Experiment 3 with *Validity* (valid vs. invalid) as within-factor, *Exposure* (yes vs. no) as a between-factor, and RTs as dependent variables to examine whether pre-exposure reduced the “reflexiveness” of attentional orienting for the 60%. We ran this additional ANOVA as we expected that top-down and bottom-up processes would cancel each other out after pre-exposure. If these two components do cancel each other out, it would lead to null results in the main 2 × 6 ANOVA, which can be challenging to interpret, therefore, the second ANOVA compared the 60% human face from Experiments 2 and 3. All *post hoc t*-tests were corrected using the (FDR) procedure.

### Results

**Figure 10:**
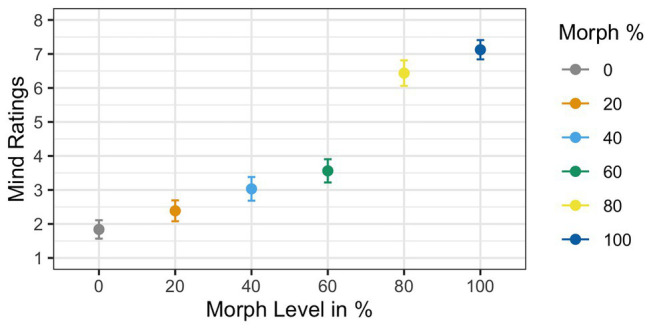
Mind ratings as a function of physical human-likeness. Analyses of the subjective mind ratings showed a main effect of *Agent Type*. Follow up tests showed that the 60% morph was rated significantly different than the 40% morph but not the 80% morph.

Two participants were excluded due to low accuracy rates (i.e., below 85%). Two additional participants had to be excluded from the mind rating analysis only, since they did not provide ratings for all six face stimuli; they were not excluded from the analysis of the gaze cueing data. Results of the ANOVA violated Mauchly’s test for sphericity [*χ* ^2^(6) = 0.29, *p* = 0.002]. Greenhouse–Geisser corrections were used to account for the assumption violation (GG *ε* = 0.73). Similar to Experiment 2, corrected estimates of the analysis of the subjective mind ratings showed a significant main effect of *Agent Type* [*F*(1,29) = 67.56, *p* < 0.001, *η_G_* ^2^ = 0.54], such that mean ratings increased as faces increased in physical humanness; see Figure 10. *Post hoc t*-tests showed that the 60% human face was significantly different than the 80% human face [*t*(29) = −7.6, *p* < 0.001, *d* = −1.41], but not the 40% human face [*t*(29) = 1.41, *p* = 0.15, *d* = 0.27]. We also tested if the average ratings for the 60% face before exposure were significantly different than the 60% face after exposure using a *t*-test. The independent samples *t*-test showed a significant reduction in ratings when participants were not pre-exposed (Experiment 1; *M* = 5.85) compared to when they were pre-exposed [Experiment 2; *M* = 3.56; *t*(64) = 4.75, *p* < 0.001, *d* = 1.16].

**Figure 11:**
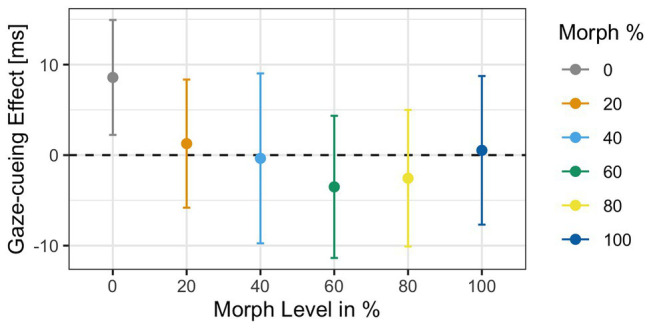
Gaze-cueing effect as a function of physical human-likeness. Analyses of the gaze cueing effect (difference in RT between valid and invalid trials) showed no differences in RT for any of the faces, validity, or interaction.

Similar to Experiment 2, accuracy rates in the gaze-cueing task were high, and only 5% were rejected due to incorrect responses (*M* accuracy = 95%, *SD* = 0.03). The results of the 2 × 6 ANOVA data showed no main effects of *Validity* [*F*(1,31) = 0.07, *p* = 0.78, *η_G_* ^2^ < 0.001] or *Agent Type* [*F*(1,31) = 0.68, *p* = 0.63, *η_G_* ^2^ = 0.002], and no *Validity* × *Agent Type* interaction [*F*(1,31) = 0.33, *p* = 0.89, *η_G_* ^2^ < 0.001]. Similar to Experiment 1, we used contrast coding on gaze cueing effects to examine differences between the 60% morph and the grand average of all the faces. The *post hoc* test showed no significant difference between gaze cueing effects elicited by 60% morph and the grand average gaze cueing effect of all the faces [*t*(31) = −0.65, *p* = 0.51, *d* = −0.12; *M*_60% face_ = −3.5 ms vs. *M*_Grand_ = 1.86 ms]; see Figure 11.

**Figure 12:**
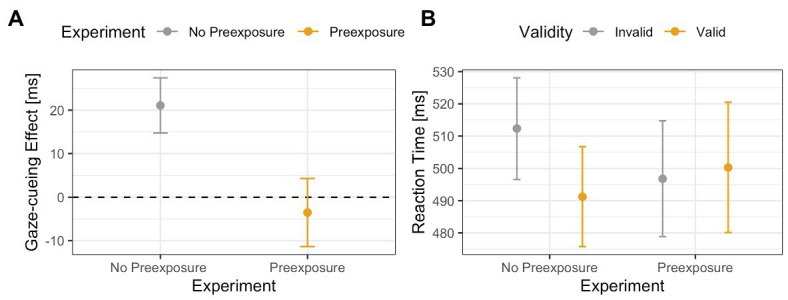
Comparison of the 60% human face in the Experiment 2 (no pre-exposure) and Experiment 3 (pre-exposure). The results show an interaction effect between exposure and validity, such that a validity effect (in this case faster reaction times toward targets are the cued location) was only present when participants were not pre-exposed to the faces. Panel **(A)** shows differences between the gaze-cueing effects of the two experiments (i.e., invalid-valid trials). Panel **(B)** shows the raw reaction times between valid and invalid trials.

The results of the previous ANOVA revealed no main or interaction effects, which suggests that for all face stimuli, and especially the 60% human stimulus, top-down and bottom-up processing worked in tandem and, therefore, canceled each other out. However, since interpreting null results can be problematic, a more active method of analysis is warranted. To do this, we compared the 60% human face from the no pre-exposure experiment to the 60% human face to test if pre-exposure to ambiguous faces does indeed increase top-down processing. Results of the 2 (*Validity*: valid vs. invalid) × 2 (*Exposure*: yes vs. no) ANOVA on RTs from the gaze-cueing task showed a trending effect of *Validity* [*F*(1,65) = 3.08, *p* = 0.08, *η_G_* ^2^ = 0.002], or *Exposure* [*F*(1,65) = 0.02, *p* = 0.89, *η_G_* ^2^ < 0.001], but a significant *Validity* × *Exposure* interaction [*F*(1,65) = 6.03, *p* = 0.01, *η_G_* ^2^ = 0.004], such that participants who were not pre-exposed to the 60% human morph showed a significant standard gaze cueing effect [i.e., significantly faster RTs on valid than invalid trials: *M*_valid_ = 491 ms vs. *M*_invalid_ = 512 ms; *t*(35) = −3.04, *p* < 0.01, *d* = −0.22], whereas participants who were pre-exposed to the faces prior to the gaze-cueing task did not show a significant standard gaze cueing effect [*M*_valid_ = 500 ms vs. *M*_invalid_ = 496 ms, *t*(31) = 0.48, *p* = 0.62, *d* = 0.03], indicating that reflexive orienting toward the cued location was significantly more under top-down control; see Figure 12.

### Discussion

The third experiment aimed to investigate whether seeing categorically ambiguous gazing stimuli is causally related to participants’ ability to top-down control attentional orienting to gaze cues in a counterpredictive gaze-cueing task. To modulate the categorical ambiguity associated with morphed face stimuli, we pre-exposed participants to the stimuli prior to the gaze-cueing task and asked questions that required participants to process the perceptual features of the faces in more detail, which has been shown to reduce categorical ambiguity and drainage of cognitive resources in previous experiments (Wiese et al., 2019). If indeed categorical ambiguity was the cause for the modulation of gaze cueing effects observed in Experiment 2, pre-exposure would be expected to alleviate this modulatory effect and restore participants’ ability to top-down control attentional orienting in a counterpredictive cueing paradigm.

As expected, the results illustrated that pre-exposing participants to the morphed stimuli restored the top-down control of attention shifting as pre-exposure reduced the reflexiveness of the attention shift for the 60% morphed image compared to the 60% morphed image from Experiment 1. Specifically, while there was a significant standard gaze cueing effect for the 60% morph in Experiment 2, such that participants were faster when reacting to targets presented at the cued vs. the likely target location, there was no significant standard gaze cueing effect for the 60% morph in Experiment 3.

## General Discussion

The current study investigated the effects of categorical ambiguity on top-down control of social attention. We hypothesized that categorically ambiguous human faces would induce cognitive conflict that is due to categorizing the faces as “human” or a “nonhuman” (Experiment 1), which negatively impacts top-down modulation of attention orienting, possibly due to drainage of cognitive resources (Experiment 2), and that pre-exposure to ambiguous stimuli embedded in a perceptual task should reduce categorical ambiguity and, in turn, positively impact top-down control of social attention (Experiment 3). The results show that, indeed, categorical ambiguity induces cognitive conflict (Experiment 1) and that the degree to which attentional orienting to gaze cues can be top-down controlled varies as a function of categorical ambiguity displayed by a gazing face, which is positively related to cognitive conflict processing (Experiment 2). Finally, by pre-exposing participants to the gazing stimuli, we were able to alleviate categorical ambiguity, which partially recovers top-down control abilities (Experiment 3).

After employing mouse tracking methods to find that the 60% human face induced cognitive conflict that is due to categorizing it as a “human” or a “nonhuman,” we used a counterpredictive gaze-cueing paradigm to show that the average differences between valid and invalid trials in the 60% human face were significantly larger compared to all the other faces combined. This illustrates how top-down modulation was least exerted for the face stimulus that was closest to the category boundary. An exploratory correlational analysis provided support for the hypothesis that cognitive conflict and gaze cueing effects (i.e., the RT differences between valid and invalid conditions) are positively correlated, such that as cognitive conflict increased (i.e., larger AUCs in mouse tracking), gaze cueing effects became more reflexive/positive. In Experiment 3, we used a pre-exposure intervention to reduce cognitive conflict related to categorical ambiguity. The results indicate that after pre-exposure, there were no longer any significant differences in gaze cueing effects between the different face stimuli as the reflexiveness of gaze cueing for the 60% morphed stimulus was significantly reduced.

The findings of these experiments are partially in line with theoretical assumptions about the nature of the UV. While the UV was originally only a theoretical construct, recent studies provide empirical evidence for its existence and shed light on its potential underlying mechanisms (Kätsyri et al., 2015; MacDorman and Chattopadhyay, 2016; Mathur and Reichling, 2016; Wiese and Weis, 2020). Two theories receiving strong empirical support are the *categorical perception hypothesis* (CPH) and the *perceptual mismatch hypothesis* (PMH): the former purports that the appearance of humanoid agents triggers a cognitive conflict when it is ambiguous whether they represent human or nonhuman entities and that conflict resolution requires cognitive resources which leads to negative emotional evaluations (Cheetham et al., 2011); the latter assumes that negative affinity associated with uncanny stimuli is caused by inconsistencies between the human-likeness levels of specific sensory signals contained in nonhuman faces, such as grossly enlarged eyes displayed on an otherwise perfectly human face (MacDorman et al., 2009). In line with the CPH, it has been demonstrated that ratings of human-likeness of morphed stimuli follow a non-linear pattern with significant changes in ratings around the category boundary (Looser and Wheatley, 2010; Cheetham et al., 2011, 2014; Yamada et al., 2013), together with increased categorization times (Cheetham et al., 2014) and negative stimulus evaluations in some studies (Burleigh et al., 2013; Yamada et al., 2013; Ferrey et al., 2015) but not in others (Looser and Wheatley, 2010; Cheetham et al., 2014; MacDorman and Chattopadhyay, 2016)^2^.

The finding that top-down control of attention orienting was negatively impacted in the first experiment for only the 60% human face can be explained in terms of the CPH. As the CPH states that categorical ambiguity reaches its peak at the category boundary, subjects would recruit more cognitive resources to categorize the face as a human or nonhuman. Therefore, less cognitive resources would be available to volitionally attend to the likely but uncued location, and we expect – and find – that the largest reduction in top-down control would be for the face at the category boundary. On the other hand, on a descriptive level, the data show that all face stimuli show some extent of reduction of top-down modulation of attentional orienting [i.e., gaze cueing effects are positive for all “morphed” stimuli (i.e., 20–80% human), but negative for the “unmorphed” stimuli (0% human and 100% human)], which would be more in line with the PMH. In other words, cognitive conflict could be induced by all the morphed images since all of them show some extent of disproportionality of physical features.

Regardless of whether cognitive conflict was caused by categorical ambiguity at a global or local feature level, the observation that cognitive conflict processing negatively impacts resource demanding processes like top-down control of attention is in line with previous studies showing that humans employ cognitive control during gaze interactions to reduce interference that is introduced by task irrelevant factors and when cognitive resources are recruited to exert control, the reflexiveness of our gaze shifts is increased in response to gaze cues. For example, when cognitive control is exerted to reduce the interference from processing emotional faces (Pecchinenda and Petrucci, 2016) or executing a cognitively demanding task (Bobak and Langton, 2015), attention shifting in response to gaze-cues is more reflexive even though top-down modulation should have reduced the reflexivity of attention shifting due to the gaze shifts being non-predictive of the target location (Bobak and Langton, 2015; Pecchinenda and Petrucci, 2016). Interestingly, this effect is also evident in other social behaviors such as head orientations and not simply gaze (Visser and Roberts, 2018). In line with the interpretation of having less cognitive resources to exert cognitive control, we show a positive correlation between gaze cueing effects (i.e., the reflexiveness of gaze) and cognitive conflict, which uses up cognitive resources (Desimone and Duncan, 1995).

Other paradigms have experienced similar effects of the negative impact of cognitive conflict from categorizing ambiguous faces on an unrelated task, which supports our interpretations. For example, in a vigilance task that measures drainage of cognitive resources, Wiese et al. (2019) found that embedding categorically ambiguous faces in the vigilance task caused cognitive resources to be drained at a faster rate compared to non-ambigious faces. More importantly, they found that pre-exposure frees up cognitive resources, showing that pre-exposure leads categorically ambiguous faces to drain less cognitive resources over time in a vigilance task (Wiese et al., 2019). The effect that categorical ambiguity influences cognitive resources is similar to the observed data that suggest that the amount of cognitive conflict that a face exerts is correlated with the reflexivity of attention orienting. This account is corroborated by both face perception literature showing that face perception is a reflexive process that does not need explicit instructions to be processed (for a review, see Palermo and Rhodes, 2007) and evidence showing that mind perception is an automatic processes that is driven by perceptual features of stimuli (Gao et al., 2010; Looser and Wheatley, 2010).

In support of our interpretation that pre-exposure allowed for cognitive conflict to be reduced, prior research has shown that merely processing stimuli in terms of their physical perceptual properties can reduce cognitive conflict (Bornstein and D’agostino, 1992; Lee, 2001) and that looking at novel stimuli for longer durations is enough to allow for more perceptual features to be processed, which results in the coactivation of multiple categories to be reduced (Li et al., 1993; Desimone and Duncan, 1995) and cognitive resources to be freed up to exert cognitive control (Desimone and Duncan, 1995; Ferrey et al., 2015). In other words, once the novelty of seeing the categorically ambiguous face is reduced due to increased exposure time, drainage of cognitive resources due to competition of categorical representations is reduced as well and more resources are available to be allocated for top-down control. This interpretation is further supported by neural data suggesting that less neurons in the inferior temporal (IT) cortex are activated as stimuli become more familiar to Rhesus monkeys when completing a delayed matching to sample (DMS) task (Li et al., 1993), which can explain why seeing an ambiguous and novel face can recruit more resources to compete for visual processing of information.

The findings of this study have several implications. First, not only did we show that cognitive conflict can reduce our ability to top-down modulate attention orienting, we also showed that resolving the conflict frees up more resources to exert cognitive control and attend to the likely location for better task performance. Although this contrasts studies that showed no effect on the modulation of gaze cueing effects when cognitive load is increased due to a secondary task (Law et al., 2010; Hayward and Ristic, 2013), it supports classical interpretations of the reflexivity of the gaze cueing effect (Friesen and Kingstone, 1998). However, it is important to note that it is possible that these gaze-cueing studies did not warrant top-down control in their design as their manipulations were considered not taxing enough to use many cognitive resources, which means that there were enough resources to be allocated for top-down modulation (Bobak and Langton, 2015). On the other hand, other studies have shown that when the gaze-cueing paradigm is in a context where a task-irrelevant influencer can affect gaze, cognitive control is warranted to inhibit the effect of the exogenous influencers, which limits the resources that allow for cognitive control to inhibit the influence of the task-irrelevant distractor (Bobak and Langton, 2015; Pecchinenda and Petrucci, 2016; Visser and Roberts, 2018). Our data are best interpreted along the latter interpretation as face processing is automatic (Palermo and Rhodes, 2007). In other words, when seeing a perceptually ambiguous face, it can serve as a distractor that limits cognitive resources from exerting top-down processing, which supports accounts that suggest continuous interactions between top-down and bottom-up processes, as opposed to classical interpretations of the gaze cueing effect (see Capozzi and Ristic, 2020, for a review on the debate between bottom-up and top-down processes of gaze-cueing).

Another implication is that mind perception is an effective modulator of gaze cueing (in line with Teufel et al., 2010; Wiese et al., 2012, 2018, 2019; Morgan et al., 2018), but that the impact of mind perception in gaze cueing depends on how mind perception is manipulated. For example, studies using belief manipulations show a positive effect on attention orienting as belief can be very effective in enhancing or decreasing social relevance (Müller et al., 2011, 2014; Wykowska et al., 2014; Caruana et al., 2015, 2017a; Fini et al., 2015; Gobel et al., 2017) and that these effects persist at a short SOA of 250 ms, which suggests that the social relevance of the gazer is determined fairly quickly (Wiese et al., 2012). However, using physical appearance as a mind perception manipulation does not have a straightforward effect on performance as (1) physical appearance and mind perception have a nonlinear relationship (i.e., UV effect) and (2) people need to first process and categorize a face before the social relevance of the face can have an effect. This observation has been shown in gaze-cueing studies that illustrate the need for longer SOAs in order for top-down modulation of facial expressions to take place (Graham et al., 2010).

We also acknowledge limitations that can be addressed in future studies. While morphed stimuli can provide an advantage in experimental control, there seems to be growing criticism in using morphed stimuli in HRI (Mathur and Reichling, 2016). While previous morph studies have generalized to real-life interactions (Kompatsiari et al., 2018), it is important to acknowledge that this may not be the case with all paradigms. Therefore, future studies should examine real robot face stimuli that vary in physical human likeness (see the ABOT database for robot faces; Phillips et al., 2018). Another possible limitation is the independence of samples across participants. While the differences between the experiments can be more informative if the same samples were presented with all three studies, it would have been difficult to tease apart the influence of the pre-exposure treatment from the duration of the experiment itself. In other words, if participants went through all three experiments, they could have been pre-exposed to the faces by simply partaking in a long study. Finally, some of our analyses employ statistical procedures that require larger sample sizes than is feasible for experimental studies. While we tried to alleviate these issues, future studies should investigate these effects using large enough samples.

While these experiments link cognitive conflict and top-down processing in a gaze-cueing study behaviorally, future studies should investigate the neurophysiological origins of this effect to disentangle top-down and bottom-up processing of gaze-cueing. For example, while Wiese et al. (2018) have shown that the ventromedial prefrontal coretex (vmPFC) is correlated with more top-down processing in a gaze-cueing study, it is unclear if variations in activation of the vmPFC is related to this cognitive conflict as other studies have suggested that the anterior cingulate cortex (ACC) is related to monitoring conflict in the brain (Botvinick et al., 2004; Umemoto et al., 2018). Future studies should also investigate if cognitive conflict varies as a function of self-other representations in the task as prior work has suggested that gaze-cueing comprises of two different processes in the brain (i.e., self-representations and other-representations; Schilbach et al., 2013). These representations allow us to either respond or engage in joint attention, which the classic gaze-cueing study does not differentiate (Caruana et al., 2017b). These representations allow us to either respond to or engage in joint attention, but as of now, it is unclear if cognitive conflict that is due to seeing ambiguous faces affects both representations.

These findings provide important considerations for the field of social robotics. For example, we illustrate the importance of understanding how cognitive processing is affected in the context of robots that try to evoke social responses from humans as we show that physical human-likeness may not be the best design choice for resource-demanding tasks executed with or in the presence of robots, since categorical ambiguity seems to be processed without being task relevant and, thus, takes away resources from the main task. Additionally, we provide a possible solution to the UV phenomenon by showing that exposure can be used as an intervention to alleviate symptoms that are due to design drawbacks.

## Data Availability Statement

The datasets presented in this study can be found in online repositories. The names of the repository/repositories and accession number(s) can be found here: https://osf.io/73pr6/.

## Ethics Statement

The studies involving human participants were reviewed and approved by Internal Review Board of George Mason University. The patients/participants provided their written informed consent to participate in this study.

## Author Contributions

EW and AM conceptualized the study. AM collected the data. AA analyzed the data. AA, EW, and AM interpreted the results and wrote the manuscript. All authors contributed to the article and approved the submitted version.

### Conflict of Interest

The authors declare that the research was conducted in the absence of any commercial or financial relationships that could be construed as a potential conflict of interest.

## Footnotes

^1^Since we created a morphing spectrum that increased in humanness from a 0% human to a 100% human in steps of 20%, the closest morph to the category boundary is the 60% human. While the 80% human is also close to the boundary, it is also much closer to the 100% human category, which could reduce any coactivation of the human and nonhuman categories.

^2^Studies examining the PMH suggest that the most negative affective evaluations are elicited by images where the mismatch between a subset of realistic (e.g., human face shape) and a subset of unrealistic image features (e.g., enlarged eyes) is maximal (Seyama and Nagayama, 2007; MacDorman et al., 2009; Mitchell et al., 2011; Mäkäräinen et al., 2014) and that maximal negative affinity does not coincide with maximal categorical uncertainty (MacDorman and Chattopadhyay, 2016).
